# Prognostic value and immunomodulatory role of DNM1L in gastric adenocarcinoma

**DOI:** 10.3389/fonc.2024.1453795

**Published:** 2024-10-24

**Authors:** Zhuo Zhao, Lingxia Li, Yan Liu, Lei Shi, Meijie Yuan, Hongshuo Shi, Qing Ji, Guobin Liu, Jian Sun

**Affiliations:** ^1^ Department of Peripheral Vascular Diseases, Shuguang Hospital, Shanghai University of Traditional Chinese Medicine, Shanghai, China; ^2^ Department of Clinical Laboratory, Shuguang Hospital, Shanghai University of Traditional Chinese Medicine, Shanghai, China; ^3^ Department of Medical Oncology, Shuguang Hospital, Shanghai University of Traditional Chinese Medicine, Shanghai, China

**Keywords:** DNM1L, gastric cancer, mitochondria, prognosis, immune infiltration

## Abstract

**Background:**

Mitochondrial fusion and fission are critical for the morphology and function of cells. DNM1L encodes dynamin-related protein 1 (DRP1), a key protein mediating mitochondrial fission, which is upregulated in a variety of cancers and is strongly associated with tumorigenesis. We aim to investigate the relationship between DNM1L and the prognosis of gastric cancer, as well as to explore the function and mechanism of DNM1L in gastric cancer (GC).

**Materials and methods:**

In this study, we analyzed the expression differences of DNM1L in gastric cancer tissues and paracancerous tissues using The Cancer Genome Atlas (TCGA) and the Gene Expression Omnibus (GEO) database. This was followed by validation through tissue microarrays. We then utilized the cohort information from these microarrays to explore the relationship between DNM1L expression and gastric cancer prognosis. Furthermore, we conducted enrichment analysis to investigate the function and mechanisms of DNM1L in gastric cancer, and lastly, we performed immune cell infiltration analysis using the CIBERSORT algorithm.

**Results:**

We discovered that the expression of DNM1L is elevated in GC tissues. TCGA data showed that the overexpression of DNM1L was positively correlated with the T-stage of GC but not with lymph node metastasis, which was also corroborated by our immunohistochemistry experiments. Based on the Kaplan–Meier curves, the high DNM1L expression was remarkably correlated with poor overall survival in patients with GC. In addition, results of COX regression analysis indicated that high DNM1L expression was an independent prognostic factor in patients with GC. Gene Ontology (GO), Kyoto Encyclopedia of Genes and Genomes (KEGG), and Gene set enrichment analysis (GSEA) showed that DNM1L was closely associated with multiple signaling pathways and immune responses. CIBERSORT analysis indicated that increased DNM1L expression may affect the infiltration of immune cells in the tumor microenvironment.

**Conclusion:**

The results of this study indicate that DNM1L is upregulated in gastric cancer (GC) and positively correlates with the T-stage and poor prognosis of GC patients, and it plays an important role in tumor immune infiltration.

## 1 Introduction

In recent decades, the overall incidence and mortality rates of gastric cancer (GC) have been steadily decreasing globally. Nevertheless, GC remains a common malignant tumor of the digestive system. According to the global cancer statistics, 1,089,000 new cases of GC were recorded in 2020, which ranked the fifth among the malignant tumors globally, and the number of deaths was 769,000, which ranked the fourth (1). *Helicobacter pylori* infection, age, smoking, alcohol consumption, and high salt intake are considered as the main risk factors for the development of GC (2). Among them, *H. pylori infection* and smoking are the first and second strongest risk factors for GC, respectively, whereas fruit and vegetable consumption are the first and second strongest protective factors against GC, respectively (3). Current treatments for GC are primarily based on surgery, neoadjuvant therapy, radiation therapy, postoperative chemotherapy, and immunotherapy (4). Most patients diagnosed for the first time already have advanced GC (5). For patients with advanced GC, the median overall survival (OS) is under 12 months (6). In addition, biomarkers, such as MSI, PD-L1, HER2, TMB, and EBV, are increasingly driving systemic therapies to improve patient survival rates (7). Therefore, active exploration of GC markers is necessary to improve patient survival and prognosis.

The mitochondria are double-membrane organelles composed of the IMM and outer mitochondrial membrane, and they are involved in biological processes (BPs) such as cellular metabolism, apoptosis, cellular signaling, reactive oxygen species production, and calcium homeostasis (8–12). The mitochondria are also dynamic organelles undergoing constant fusion and fission (13, 14). Mitochondrial fusion and fission are related to the survival and development of tumor cells (15). DNM1L encodes dynamin-related protein 1 (Drp1), which belongs to the GTPase dynamin superfamily (16), a key protein mediating mitochondrial fission (17). Therefore, changes in DNM1L expression are closely associated with tumor generation and progression. Studies have shown that DNM1L expression is elevated in lung adenocarcinoma, hepatocellular carcinoma, breast cancer, and head and neck cancer, and it is correlated with poor patient prognosis (18–21). However, the expression profile and prognostic value of DNM1L in GC and its relationship with clinical features and immune infiltration remain unknown.

In this study, the differences in DNM1L expression in GC tissues and normal tissues adjacent to the cancer were analyzed by using the Cancer Genome Atlas Project (TCGA) and GEO databases, and the correlation between DNM1L and invasive GC metastasis was assessed. In addition, the correlation between the protein expression of DNM1L and the clinical data and survival outcomes of patients with GC was analyzed by immunohistochemical experiments. The correlation between DNM1L and immune infiltration was also investigated using the CIBERSORT algorithm. The results of this study will reveal the important role of DNM1L in GC and suggest a potential relationship between DNM1L and tumor immune infiltration.

## 2 Materials and methods

### 2.1 Gene expression analysis

Transcriptome RNA-seq data of 446 STAD cases (36 normal and 410 tumor) and corresponding clinical information were downloaded from the Cancer Genome Atlas Project (TCGA) database (https://www.cancer.gov/ccg/research/genome-sequencing/tcga). The gene expression profiles of the GSE118916, GSE54129, and GSE66229 datasets were downloaded from the National Center for Biotechnology Information Gene Expression Omnibus (GEO) database (https://www.ncbi.nlm.nih.gov/geo/). The gene expression of DNM1L was subjected to pan-cancer analysis using the Gene_DG module of the TIMER 2.0 (http://timer.cistrome.org/) database, and the visualization results were output. In comparing the difference in DNM1L expression in normal and tumor tissues, the Wilcoxon rank-sum test was performed on the gene expression profile data downloaded from the TCGA and GEO databases using the ggpubr package in R language (version 4.3.1), and differences at *P* < 0.05 were considered statistically significant.

### 2.2 Clinical specimen

The gastric tissue microarrays (HStmA180-Su30) were provided by Outdo Biotech (Shanghai, China). The HStmA180-Su30 contained 97 GC tissue samples and 83 corresponding paracancerous normal tissue samples. After screening, 96 GC tissue samples and 83 corresponding paracancerous normal tissue samples were included in the cohort. Detailed clinicopathological characteristics of the cohorts were also provided, which included gender, age, tumor size, tumor differentiation, T stage, N stage, metastasis and expression of some tumor markers such as HER2, AFP, CEA, CA199, CA153, CA125, CA50 and *Helicobacter pylori* infection. Ethical approval for the tissue microarray study was obtained from the Clinical Research Ethics Committee of Outdo BioTech (Shanghai, China).

### 2.3 Immunohistochemical manual quantification

The immunohistochemistry kit was purchased from Maixin Bio (Fuzhou, China). Anti-DNM1L antibody (12957-1-AP; 1:500) for immunohistochemistry was purchased from ProteinTech Group, Inc. The tissue microarray slide was incubated with peroxidase blocking reagent for 10 minutes, washed three times with PBS for 3 minutes, blocked with rabbit serum at room temperature for 60 minutes, and incubated overnight with antibodies at 4 °C. Subsequently, the tissue microarray slide was washed three times with PBS, and the second antibody conjugated with HRP was incubated under RT for 10 minutes. The tissue microarray slide was washed again with PBS, developed with diaminobenzidine solution, and restrained with hematoxylin. The immunohistochemical results were scored by two pathologists in accordance with the German semi-quantitative statistical method for the degree of staining. The overall percentage of positively stained cells and the intensity of staining were used to reflect the level of protein expression. The percentage of positively stained cells was scored on a scale of 0–4: <5% as 0, 5%–25% as 1, 25%–50% as 2, 51%–75% as 3, and >75% as 4. Staining intensity was scored in accordance with the presence and depth of cellular staining in the sections: 0 point for no cellular staining, 1 point for light-yellow staining, 2 points for yellow staining, and 3 points for dark-yellow/brown staining. The combined percentage of positive staining and overall staining intensity scores was used to describe the level of protein expression: negative (0–2), + (3–5), ++ (6–8), and +++ (9–12). Furthermore, all tissues were categorized into low-expression groups (− or +) and high-expression groups (++ and ++++) (22).

### 2.4 Association of DNM1L expression with various clinicopathological features and prognosis

In exploring the relationship between DNM1L expression and clinicopathological features, first, the clinical data downloaded from the TCGA database were used, which were subjected to the Wilcoxon rank-sum test by the ggpubr package in the R language, and *P* < 0.05 was considered statistically significant. Next, the clinical data were divided into high and low groups in accordance with the protein expression level of DNM1L by our immunohistochemical scoring, and the difference between the high and low groups of DNM1L was evaluated by performing a Chi-square test for the high and low groups of DNM1L using SPSS (version 26). *P* < 0.05 was considered statistically significant.

### 2.5 Survival analysis

Survival analysis was performed using the survival package in R. The Kaplan–Meier survival curves were plotted using detailed survival data and DNM1L immunohistochemistry scores of 96 cases, and the log-rank test was performed for statistical significance, with *P* < 0.05 indicating statistical significance. One-way COX regression analysis was used to evaluate the relationship between clinical indicators (age, gender, TNM stage, tumor size, degree of differentiation, *H. pylori* infection, and DNM1L protein expression) and survival outcomes (OS, OS time) in 96 cases. In addition, screening criteria were used, and differences at *P* < 0.05 were retained for multifactorial Cox regression. In multifactorial Cox regression analysis, *P* < 0.05 was considered statistically significant.

### 2.6 GeneMANIA analysis

The GeneMANIA database (https://genemania.org/) was primarily used for function prediction of genes. In addition, genes with similar functions to DNM1L were identified using the rich genomic and proteomic data of the GeneMANIA database to construct a gene network that interacts with DNM1L (23).

### 2.7 STRING analysis

The STRING database (https://cn.string-db.org/) is a comprehensive database that plays an important role in protein–protein interaction (PPI) network research. This database was used to construct the DNM1L PPI network.

### 2.8 GEPIA analysis

The GEPIA database (http://gepia.cancer-pku.cn/) is a gene expression analysis platform based on TCGA data, which allows for tumor type analysis, single-gene analysis, and multi-gene analysis. We conducted a correlation analysis on genes that might be related to DNM1L using the Correlation Analysis module of the GEPIA database.

### 2.9 Differential, GO, and KEGG analyses

First, using the DEseq2 package in R, the TPM data of DNM1L expression downloaded from the TCGA database were used to divide the 410 tumor samples into high and low groups in accordance with the median criterion. Then, the count data downloaded from the TCGA database were used to perform differential analysis in accordance with the criterion of the DNM1L high and low groups. In addition, clusterProfiler in R was used to perform differential analysis, and the clusterProfiler package was used to perform Gene Ontology (GO) and Kyoto Encyclopedia of Genes and Genomes (KEGG) enrichment analyses for differentially expressed genes with |log2FoldChange|> 0.6 and *P* < 0.05. GO terms and KEGG pathways with a *P*-value of < 0.05 were considered significantly different.

### 2.10 Gene set enrichment analysis

The Hallmark and C7 gene set v7.0 collections were downloaded from the Molecular Characterization Database as target sets, and the results of differential analysis in GO and KEGG enrichment analyses were subjected to GSEA using the clusterProfiler package in R. Only gene sets with NOM *P* < 0.05 and FDR q < 0.25 were considered significant.

### 2.11 Immune infiltration analysis

First, the abundance of TIICS in GC samples from the TCGA database was estimated using the CIBERSORT algorithm in R language (24). Then, 22 tumor-infiltrating immune cells were divided into high and low groups in accordance with the median of the expression data of DNM1L downloaded from the TCGA database, and the differences of these cells were analyzed using the Wilcoxon rank-sum test. In addition, a *P*-value less than 0.05 indicated statistical significance. Finally, the correlation between each type of immune cells and DNM1L expression was calculated, and the results with a *P*-value less than 0.05 were retained.

### 2.12 Statistical analysis

R 4.3.1 and SPSS 26.0 were used as the main tools for statistical analysis and graphical presentation. The Wilcoxon rank-sum test was performed on the gene expression profile data and clinical data downloaded from the TCGA and GEO databases, respectively, using the plyr package of the R language. Then, the results were visualized by using the ggpubr package. The correlation between the DNM1L expression levels and clinical characteristics of patients was analyzed by the Chi-square test using SPSS. Survival analysis was performed using the survival package in R. First, the Kaplan–Meier survival curves were calculated from the survival data, and significance was determined by the log-rank test. The results were visualized using the survminer package. Then, one-way and multifactorial COX regression analyses were performed on the clinical data, and the results were visualized using the forestplot package. The abundance of TIICS in GC samples was calculated using the CIBERSORT algorithm in R. Differential and correlation analyses were performed on the expression of DNM1L and 22 tumor-infiltrating immune cells. Significance was determined using the Wilcoxon rank-sum test for differential analysis, and correlation analysis was performed using the Spearman’s rank correlation analysis to determine significance. The results were shown in the ranges of *P < 0.05, **P < 0.01, ***P < 0.001, and ****P < 0.0001, which were considered statistically significant.

## 3 Results

### 3.1 DNM1L is highly expressed in GC tissues

**Figure 1:**
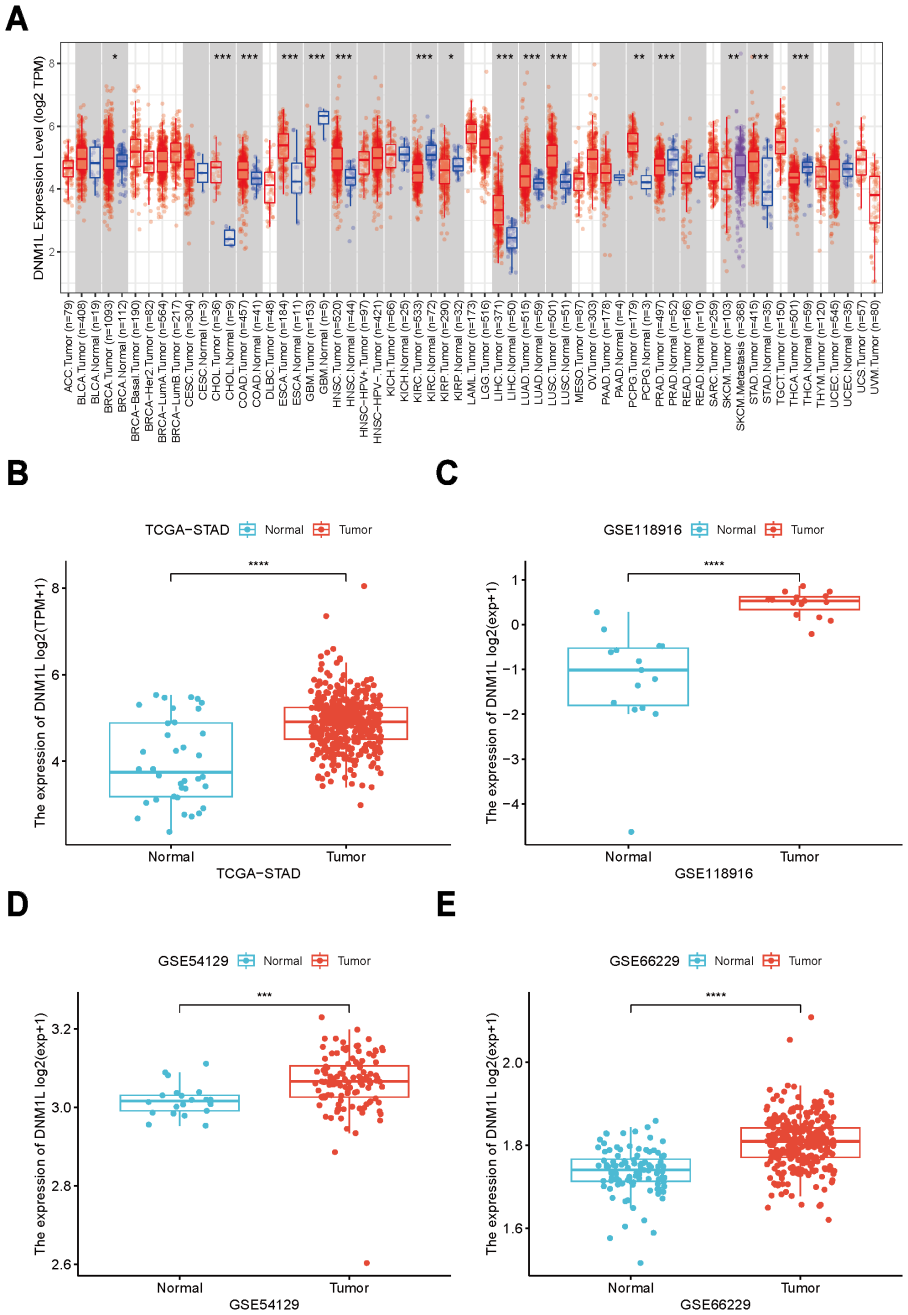
Expression levels of DNM1L in GC tissues. **(A)** Expression levels of DNM1L in various types of cancers based on the TIMER2.0 database. **(B)** Expression differences of DNM1L in GC and paracancerous tissues in the TCGA database. **(C–E)** Expression differences of DNM1L in GC and paracancerous tissues in three different datasets based on the GEO database. *P < 0.05, **P < 0.01, ***P < 0.001, ****P < 0.0001.

In assessing the involvement of DNM1L in GC, the Tumor Immunity Estimation Resource (TIMER2.0), the TCGA project database, and the GEO database of the National Center for Biotechnology Information were used to perform comprehensive analysis of whether DNM1L showed different expression levels in patients with GC. First, the overall assessment of DNM1L expression in a variety of common tumors was performed using the TIMER 2.0 online database (**Figure 1A**). DNM1L expression was significantly higher in BRCA, CHOL, COAD, ESCA, HNSC, LIHC, LUAD, LUSC, PCPG, and STAD than in paraneoplastic tissues, and it exhibited low levels in GBM, KIRC, KIRP, PRAD, SKCM, and THCA. In the TCGA database, the expression level of DNM1L was significantly higher in GC tissues than in paracancerous tissues (*P* < 0.05, **Figure 1B**). Using the GEO database to validate the TCGA results, the GSE118916, GSE54129, and GSE66229 datasets showed that the expression level of DNM1L was significantly higher in GC tissues than in paracancerous gastric tissues (**Figures 1C–E**).

### 3.2 Relationship between the protein expression of DNM1L and clinicopathological parameters

**Figure 2:**
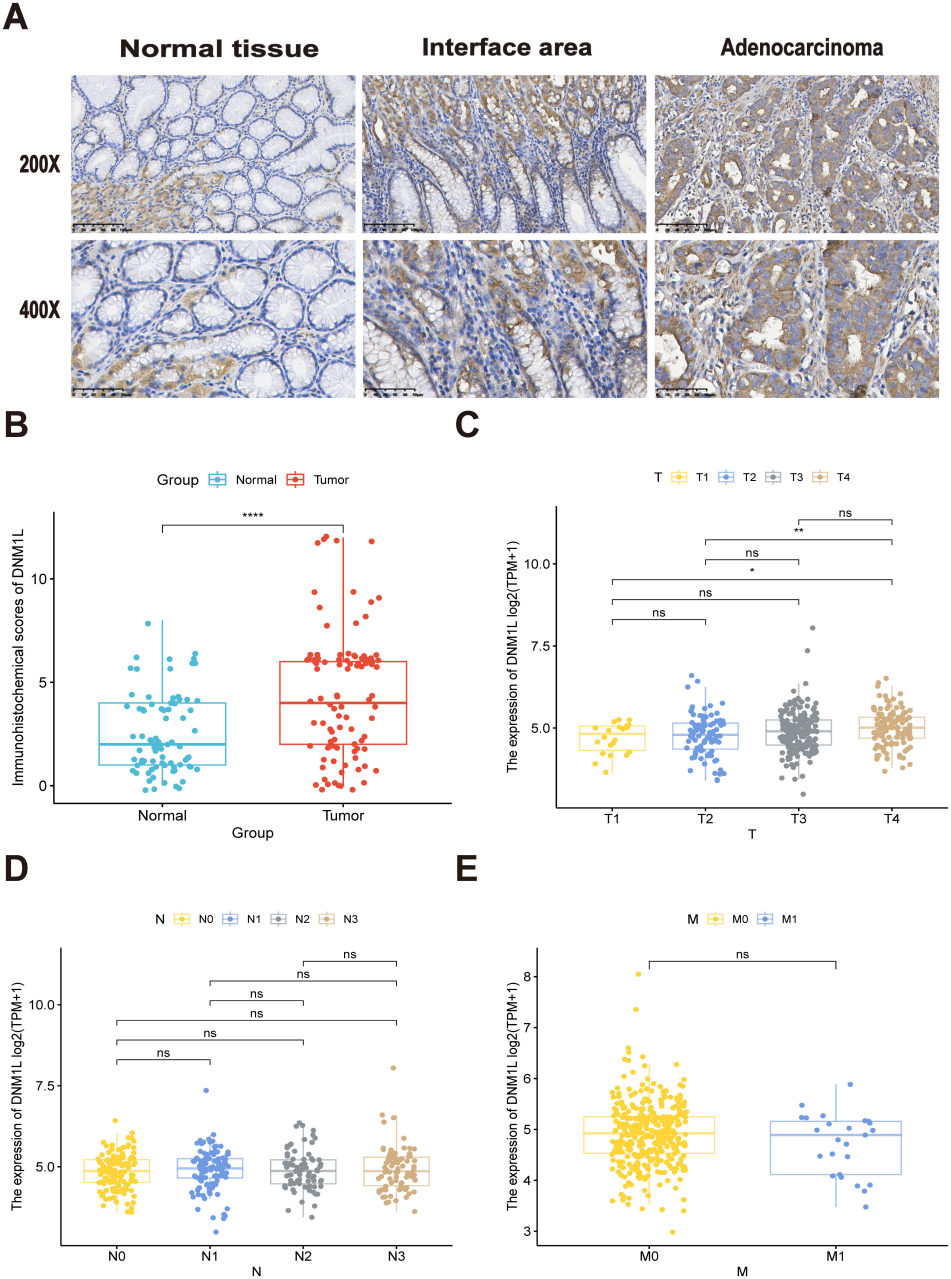
Representative results of immunohistochemical staining and correlation of DNM1L expression with TNM stage of GC. **(A)** Representative images of DNM1L immunohistochemical staining of 180 human patients with GC. **(B)** Immunohistochemical scores of GC and normal tissues adjacent to carcinoma. **(C–E)** DNM1L based on the TCGA database of GC tissues with different TNM stages and expression differences. *P < 0.05, **P < 0.01, ****P < 0.0001.

The immunohistochemical staining results further confirmed the upregulation of DNM1L in GC samples. Even in the junctional region, DNM1L was still overexpressed in the tumor region as compared with the normal region (**Figure 2A**). The expression level of DNM1L in GC tissues was higher than that in their neighboring normal tissues (*P* < 0.05, **Figure 2B**). To comprehensively understand the correlation and potential mechanisms of DNM1L expression, the relationship between the expression of DNM1L and the clinical characteristics of GC samples was investigated. The results of the analysis of TCGA clinical data shown in **Figures 2C–E** indicated that overexpression of DNM1L was positively correlated with the T-stage of GC (*P* < 0.05) but not with lymph node metastasis. The correlation between our clinical data results and DNM1L expression is shown in **Table 1**, which further verified that DNM1L overexpression was positively correlated with T-stage (*P* < 0.05). Furthermore, DNM1L overexpression was negatively correlated with prognosis in patients with GC (*P* < 0.001).

**Table 1:** Correlation of DNM1L expression with clinicopathologic features in GC tissues.

Clinico-pathologic features	[ALL]	high	low	p.values
	*N=96*	*N=47*	*N=49*	
Age				0.095
<65	65 (67.7%)	28 (59.6%)	37 (75.5%)	
≥65	31 (32.3%)	19 (40.4%)	12 (24.5%)	
gender				0.405
female	23 (24.0%)	13 (27.7%)	10 (20.4%)	
male	73 (76.0%)	34 (72.3%)	39 (79.6%)	
T				0.030*
T1+T2	26 (27.1%)	8 (17.0%)	18 (36.7%)	
T3+T4	70 (72.9%)	39 (83.0%)	31 (63.3%)	
N				0.285
N0+N1	40 (41.7%)	17 (36.2%)	23 (46.9%)	
N2+N3	56 (58.3%)	30 (63.8%)	26 (53.1%)	
M				0.705
M0	83 (86.5%)	40 (85.1%)	43 (87.8%)	
M1	13 (13.5%)	7 (14.9%)	6 (12.2%)	
degree				0.923
low	49 (53.8%)	25 (54.3%)	24 (53.3%)	
middle+high	42 (46.2%)	21 (45.7%)	21 (46.7%)	
*H. Pylori* infection				0.559
negative	37 (50.7%)	20 (54.1%)	17 (47.2%)	
postive	36 (49.3%)	17 (45.9%)	19 (52.8%)	
fustat				<0.001***
alive	30 (31.2%)	6 (12.8%)	24 (49.0%)	
dead	66 (68.8%)	41 (87.2%)	25 (51.0%)	

*P < 0.05, ***P < 0.001.

### 3.3 DNM1L overexpression correlates with poor prognosis in gastric cancer patients

**Figure 3:**
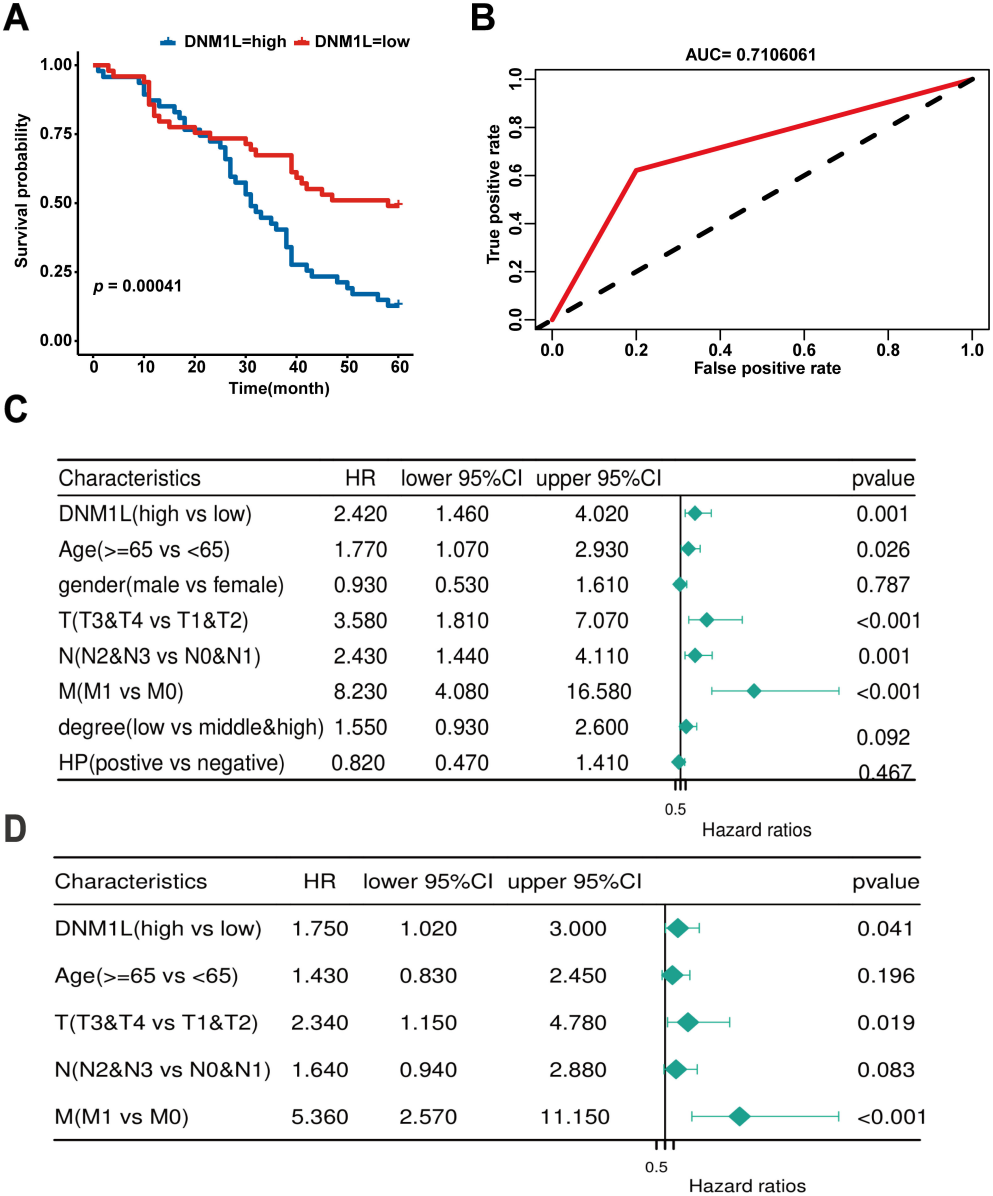
Prognostic value of DNM1L expression in patients with GC. **(A)** Correlation between DNM1L expression and OS was analyzed by using the Kaplan–Meier curves **(B)** ROC curves assessing the feasibility of the Kaplan–Meier curves. **(C)** Relationship between DNM1L expression and clinicopathological characteristics in patients with GC analyzed by one-way COX regression. **(D)** Relationship between DNM1L expression and clinicopathological features in patients with GC analyzed by multifactorial COX regression.

The relationship between DNM1L expression and the prognosis of patients with GC was observed by plotting KM curves, and patients with a higher DNM1L expression level had significantly poor OS (*P* = 0.00041, **Figure 3A**). The predictive efficacy of high and low DNM1L expression for the prognosis of patients with GC was analyzed using the subject’s work characteristic curve (ROC, **Figure 3B**). The area under the curve of DNM1L was 0.7106061, indicating that high and low DNM1L expression levels indicated high sensitivity and specificity for the diagnosis and prognosis of patients with GC.

After KM curve analysis, univariate Cox regression analysis was used to assess the relationship between DNM1L expression and prognosis of patients with GC. The results indicated that higher DNM1L expression (HR = 2.420, 95% CI: 1.460–4.020, *P* = 0.001), ≥65 years (HR = 1.770, 95% CI: 1.070–2.930, *P* = 0.026), T3 and T4 stages (HR = 3.580, 95% CI: 1.810–7.070, *P* < 0.001), N2 and N3 stages (HR = 2.430, 95% CI: 1.440–4.110, *P* = 0.001), and M1 stage (HR = 8.230, 95% CI: 4.080–16.580, *P* < 0.001) were significantly associated with poor prognosis in patients with GC (**Figure 3C**). Next, the results of multifactorial COX regression showed that higher DNM1L expression (HR = 2.420, 95% CI: 1.460–4.020, *P* = 0.001), T3 and T4 staging (HR = 3.580, 95% CI: 1.810–7.070, *P* < 0.001), and M1 stage (HR = 8.230, 95% CI: 4.080–16.580, *P* < 0.001) remained significantly associated with poor prognosis in patients with GC, and higher DNM1L expression level was an independent prognostic biomarker for patients with GC (**Figure 3D**).

### 3.4 Screening key genes from DNM1L interaction networks

**Figure 4:**
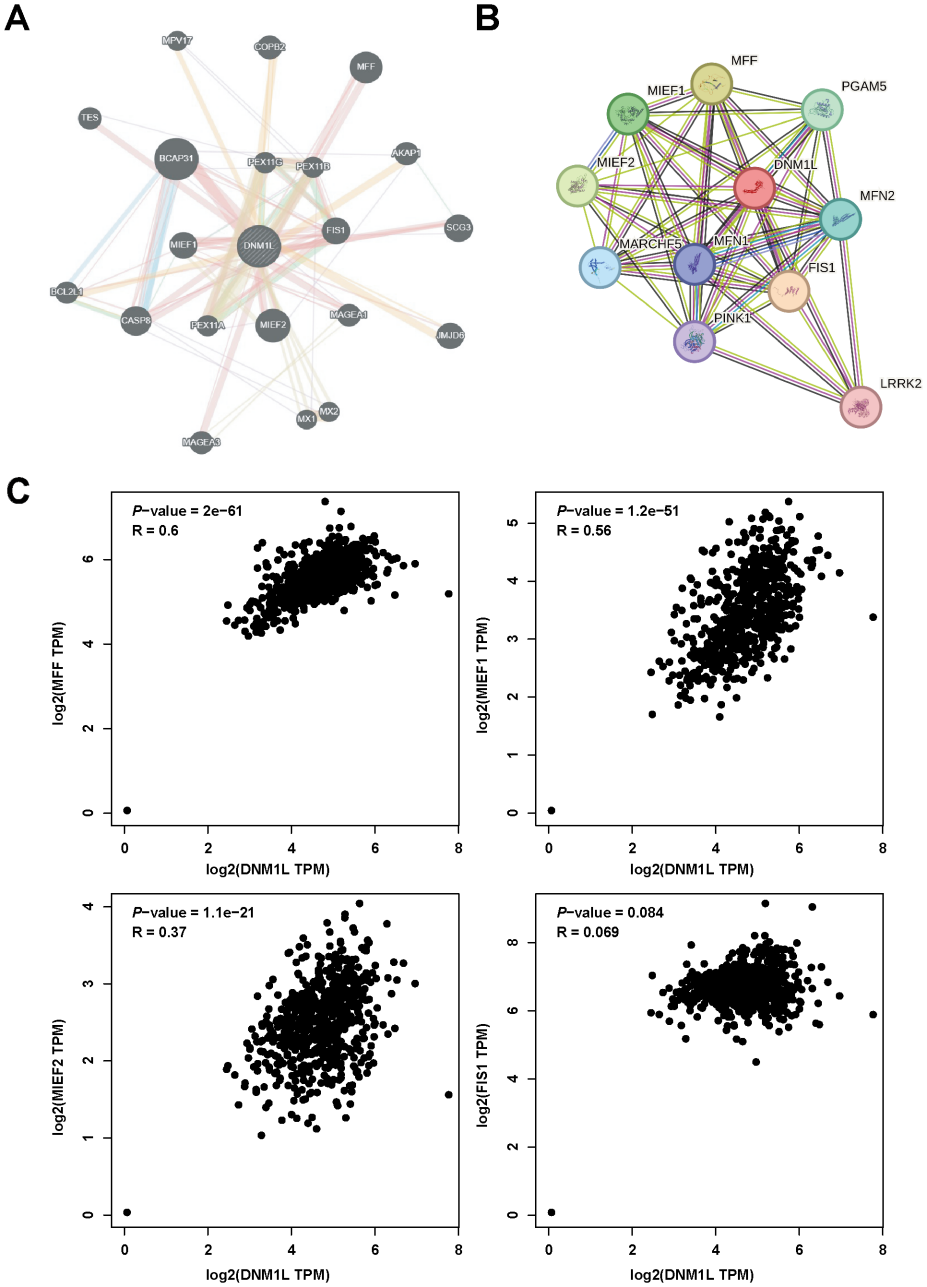
DNM1L-related gene analysis. **(A)** Construction of the DNM1L gene–gene interaction network using GeneMANIA. **(B)** PPI network of DNM1L generated using STRING. **(C)** Scatterplot of the correlation between the expression of DNM1L and that of MFF, MIEF1, MIEF2, and FIS1 in GC analyzed by using the GEPIA database.

Gene–gene networks interacting with DNM1L were constructed using the GeneMANIA database. The results showed that 20 genes, including BCAP31, MIEF2, and MFF, were most associated with DNM1L (**Figure 4A**). Protein–protein interaction networks interacting with DNM1L in *Homo sapiens* were obtained using the STRING database. DNM1L served as the center and contained 50 edges and 11 nodes. As shown in the figure, the genes interacting with DNM1L include MFF, M1EF1, and FIS1 (**Figure 4B**). By integrating the results of the abovementioned two databases, four genes were found to be identical, namely, MFF, MIEF1, MIEF2, and FIS1. Accordingly, we further evaluated the correlation between DNM1L and these four genes using the GEPIA database. As shown in **Figure 4C**, in GC, DNM1L expression was strongly correlated with the expression of MFF, MIEF1, MIEF2, and FIS1. The correlation coefficients for each of these genes with DNM1L are all greater than 0, indicating a positive correlation between DNM1L expression and the expression of MFF, MIEF1, MIEF2, and FIS1.

### 3.5 Differential analysis of DNM1L expression and GO and KEGG analyses

**Figure 5:**
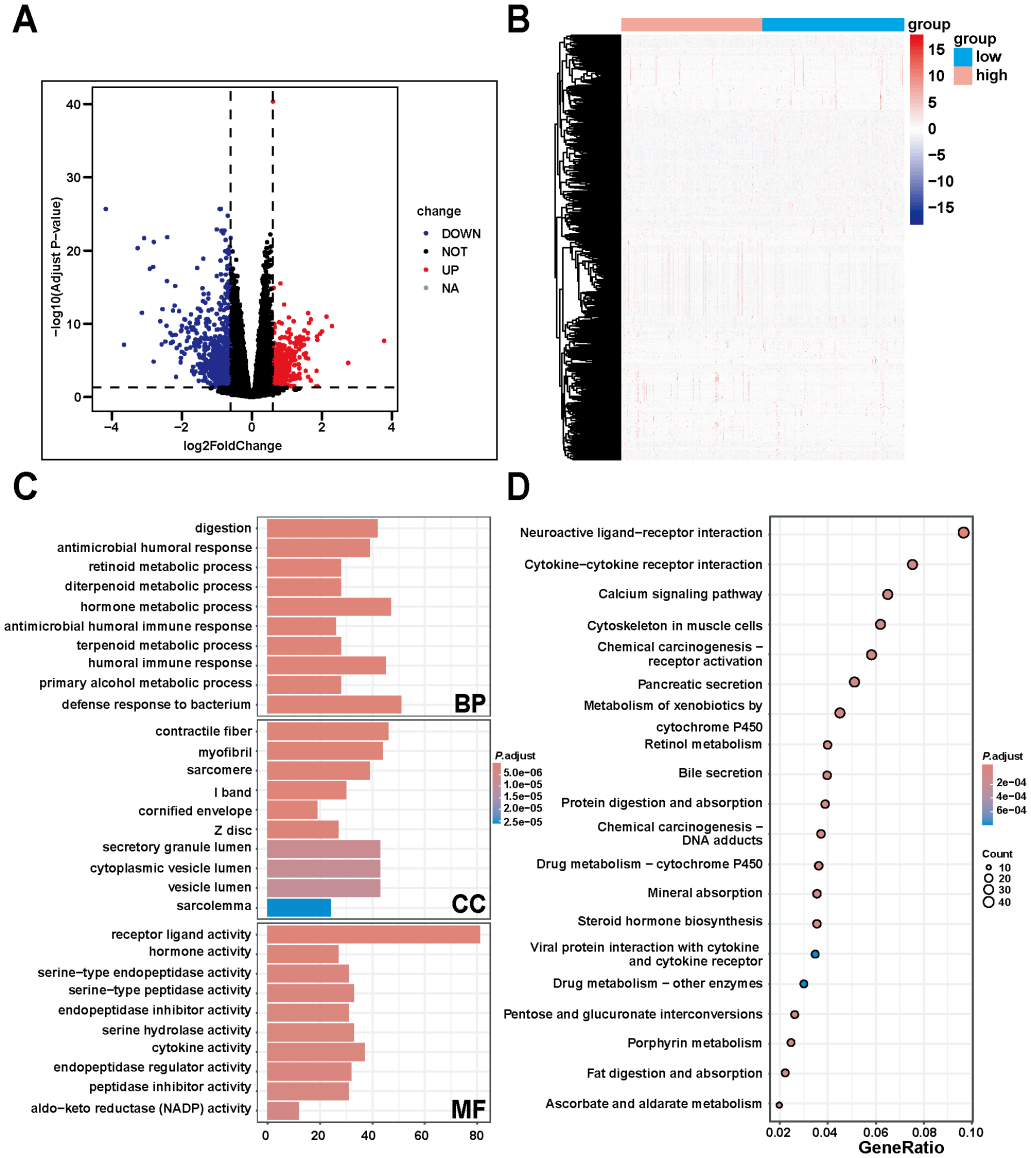
Differential, GO, and KEGG enrichment analyses based on TCGA gastric cancer data. **(A)** Differentially expressed genes of DNM1L obtained by differential analysis. **(B)** Heatmap constructed using TCGA gastric cancer data showing the differentially expressed genes positively and negatively correlated with DNM1L expression. **(C)** DNM1L gene set ranked among the top 10 enriched terms in BP, CC, and MF in GO enrichment analysis. **(D)** DNM1L gene sets ranked in the top 20 pathways in KEGG enrichment analysis.

The expression data of DNM1L downloaded from the TCGA database were used for differential analysis, and the results of the differential analysis were screened using the following criteria: |log2FoldChange|> 0.6 and *P*< 0.05. A total of 414 upregulated genes, 760 downregulated genes, and 18,275 genes with no significant changes were obtained (**Figures 5A, B**). In exploring the biological functions and pathways of DNM1L, GO enrichment analysis was performed on the screened differential genes, and the results showed the top 10 most significant biological effects under each item of BP, cellular composition (CC), and molecular function (MF). As shown in **Figure 5C**, the horizontal coordinates represent the number of genes enriched in the item; vertical coordinates represent the name of the biological effect, and the color represents the corrected *P*-value, wherein the redder the color, the stronger the difference. The analysis results indicated that in BP, DNM1L was enriched during metabolic processes particularly digestion, immune response, antimicrobial humoral response, retinoid metabolic process, diterpene metabolic process, hormone metabolic process, and antimicrobial peptide-mediated humoral immune response. In CC, DNM1L was primarily associated with skeletal muscles, and the terms enriched were contractile fibers, myogenic fibers, myonuclei, I-bands, and cuticular envelope. In MF, the enrichment results indicated that DNM1L was involved in the inhibition and regulation of receptor ligands, hormones, cytokines, and a variety of enzyme activities, such as receptor ligand activity, hormone activity, serine-type endopeptidase activity, endopeptidase inhibitor, and cytokine activity.

Subsequent KEGG enrichment analysis yielded a bubble map of the top 20 pathways of the DNM1L differential gene set (**Figure 5D**); the larger the bubble and the redder the color, the more the pathway was affected by the DNM1L differential gene set. The analysis indicates that DNM1L is mainly enriched in pathways related to tumorigenesis and metabolic pathways, such as chemical carcinogenesis - receptor activation, metabolism of xenobiotics by cytochrome P450, retinol metabolism, chemical carcinogenesis - DNA adducts, and drug metabolism - cytochrome P450.

### 3.6 GSEA of DNM1L-related signaling pathways

**Figure 6:**
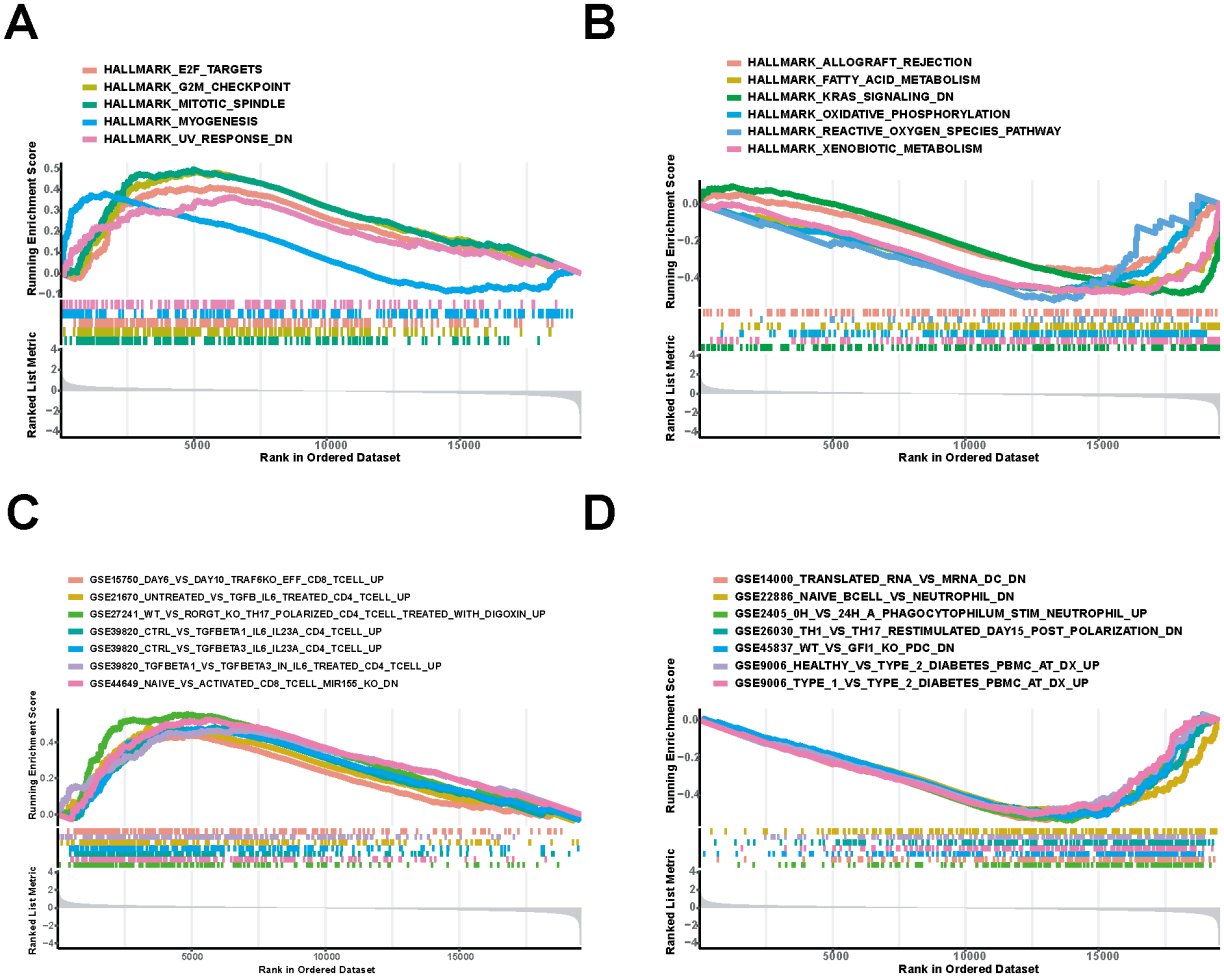
Pathways most involved in the DNM1L differential gene set obtained by GSEA. **(A)** Gene sets enriched in the HALLMARK set for the DNM1L high-expression group. **(B)** DNM1L low-expression group in the HALLMARK set of the enriched gene sets. **(C)** Set of genes enriched during C7 collection by the DNM1L high-expression group. **(D)** Gene sets enriched during C7 collection by the DNM1L low-expression group.

DNM1L was divided into high and low-expression groups for GSEA using the median expression of DNM1L as the criterion to explore the molecular mechanism of DNM1L in GC, and 11 pathways were enriched in the HALLMARK set. In addition, the genes in the high-expression group of DNM1L were mainly enriched during cellular mitosis, such as genes coding for EF2 targets, genes involved in the G2/M checkpoint, and genes involved in the G2/M checkpoint and spindle assembly (**Figure 6A**). For the DNM1L low-expression group, genes were enriched during immune-related activities and metabolic pathways, including graft rejection, fatty acid metabolism, oxidative phosphorylation, and aerobic metabolism (**Figure 6B**). For the MSigDB-defined C7 set, which served as the immune gene set, 380 pathways were enriched. Multiple immune function gene sets were enriched in the DNM1L high and low-expression groups (**Figures 6C, D**). These results indicate that DNM1L is involved in the regulation of multiple molecular signaling pathways in GC progression, especially in immune and metabolism-related pathways. Considering that GSEA uses genome-wide expression profiles obtained by sequencing or microarray analysis, the thresholds for differential genes need not to be specified, which can be used as a complement to the GO and KEGG results.

### 3.7 Correlation analysis of DNM1L expression and tumor-infiltrating immune cells

**Figure 7:**
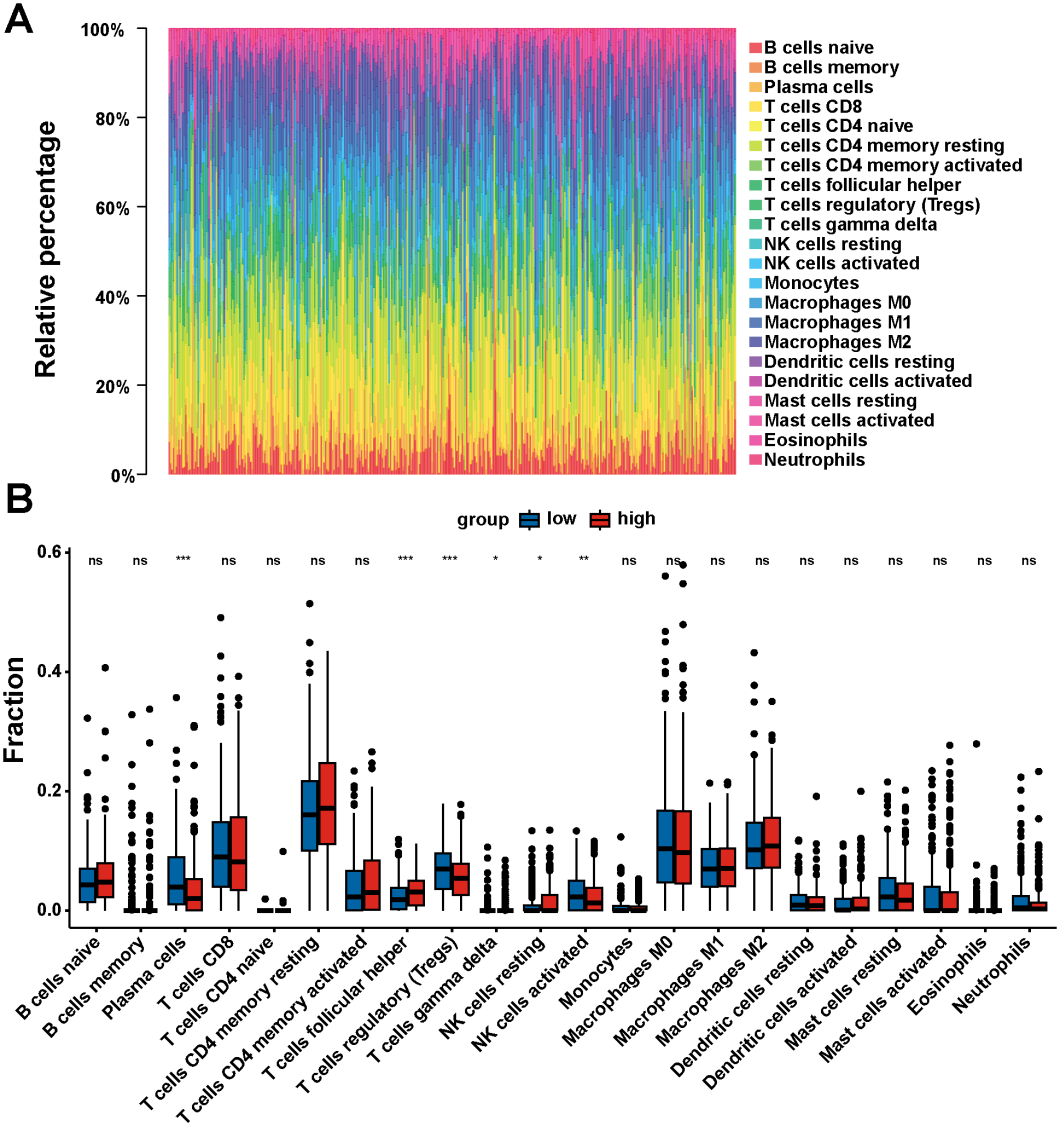
Correlation of DNM1L expression with tumor-infiltrating immune cells. **(A)** Rainbow plot showing the proportion of 22 tumor immune-infiltrating cells in STAD tumor samples. **(B)** Violin plot showing the proportion of 22 types of immune cells differentiated in the DNM1L low expressing group and high expressing STAD tumor samples. *P* < 0.05 indicates statistical significance using Wilcoxon rank-sum for significance test. *P < 0.05, **P < 0.01, ***P < 0.001.

**Figure 8:**
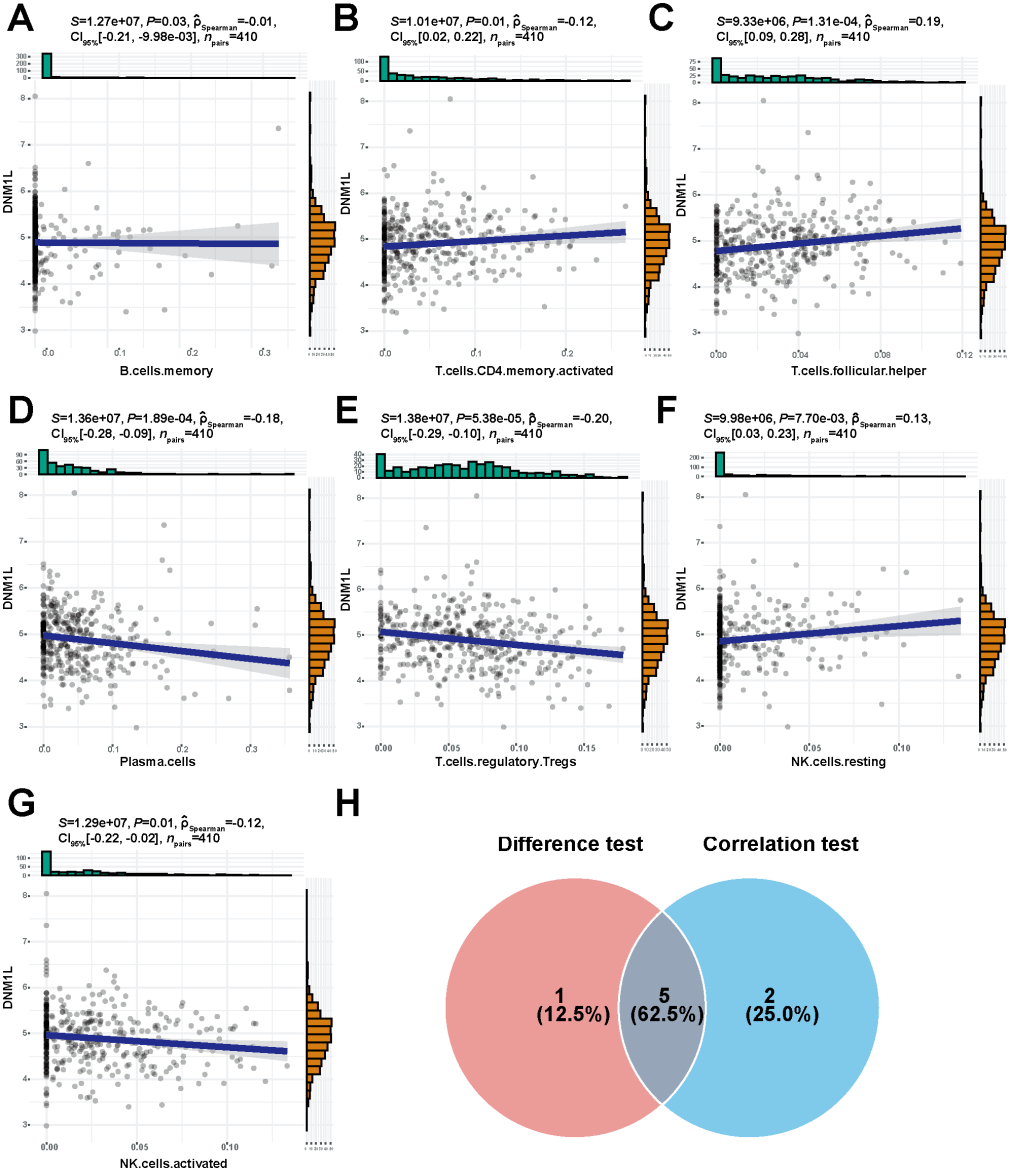
High expression of DNM1L is associated with seven tumor-infiltrating immune cells. **(A–G)** Scatter plot showing the correlation between DNM1L expression and the proportion of seven tumor-infiltrating immune cells. The *P*-values for all seven tumor immune-infiltrating cells presented in the graphs were <0.05. The line in each graph indicates the convergence of the correlation of DNM1L expression with the proportion of immune cells, and a correlation test was performed using Spearman’s correlation coefficient. **(H)** Wayne plots showing DNM1L expression versus tumor-infiltrating immune cells for the five tumor-infiltrating immune cells taken as intersections of differential and correlation analyses.

The proportion of tumor-infiltrating immune cells was analyzed using the CIBERSORT algorithm to explore the role of DNM1L in the tumor microenvironment, and 22 types of immune cell profiles in STAD samples were constructed from the DNM1L expression data downloaded by TCGA (**Figure 7A**). Differential analysis showed that six tumor-infiltrating immune cells were associated with DNM1L expression (**Figure 7B**), whereas correlation analysis showed that seven tumor-infiltrating immune cells were associated with DNM1L expression (**Figures 8A–G**). The intersection of differential and correlation analyses was recorded to obtain five types of tumor immune-infiltrating cells, among which two types of cells were positively correlated with DNM1L expression, including follicular, helper, NK, resting, and T cells (**Figure 8H**). Moreover, three types of tumor-infiltrating immune cells were negatively correlated with the expression level of DNM1L, namely, plasma cells, T regulatory cells (Tregs), NK cells, and activated cells. Therefore, DNM1L affects the immunoreactivity of the tumor microenvironment.

## 4 Discussion

The mitochondria are dynamic organelles that are constantly undergoing fusion and division, which determine mitochondrial morphology and are critical to mitochondrial function (25). Increasing evidence shows that mitochondrial fusion and division are closely associated with tumor cell development and that mitochondrial division is increased in a variety of human cancer cells (15). Mitochondrial division first involves a contraction event, marked by the contact between the mitochondria and the endoplasmic reticulum mediated by the actin–myosin cytoskeleton, followed by the recruitment of Drp1 to the fission site, a process mediated primarily by the four endosomal membrane proteins, namely, Fis1, Mff, MiD49, and MiD51 (also known as MIEF1 and MIEF2). Then, Drp1 was recruited to the fission site and assembles into oligomeric rings, which in turn are hydrolyzed by GTP to drive conformational changes that further contract these rings to drive mitochondrial division (26, 27). In addition, Drp1, which is encoded by DNM1L, is a key protein that mediates mitochondrial division (17). Therefore, the changes in DNM1L are closely related to tumor development.

Studies have shown that Drp1 expression is upregulated in a variety of cancers and that Drp1 is associated with several metabolic pathways leading to cancer, including the PI3K-Akt, MAPK/ERK1/2, mTOR, AMPK, and Wnt/β-cyclin pathways (28). For example, Drp1 overexpression induces mitochondrial dysfunction, followed by cytoplasmic mtDNA stress and subsequent activation of the cGAS-STING pathway to promote esophageal squamous cell carcinoma progression (29). Drp1 promotes fatty acid–induced activation of Wnt signaling through β-catenin acetylation to influence colon cancer tumorigenesis (30). Moreover, mitochondrial plasticity can be targeted by attenuating or inhibiting Drp1 expression, thereby reducing breast cancer metastasis to the brain (31). Drp1 is also highly expressed in pancreatic cancer and is remarkably associated with poor prognosis in patients with pancreatic cancer, and Drp1 promotes the growth and metastasis of pancreatic cancer cells by promoting aerobic glycolysis (32). The inhibition of Drp1 reduces mitochondrial fission to regulate BRAFV600E-mediated colorectal cancer progression, which in turn regulates glycolytic metabolism required for tumorigenesis (33). Therefore, Drp1 could be a next-generation target for the treatment of cancer and mitochondrial dysfunction diseases. However, the expression profile and prognostic value of Drp1 in GC have not been fully explored, and the relationship between Drp1 and GC immune infiltration remains unclear.

In this study, bioinformatic analysis was performed using four sets of transcriptome sequencing data of GC tissues and paracancerous tissues downloaded from the TCGA and GEO databases. The results indicated that the expression of DNM1L in GC tissues was significantly higher than that in paracancerous tissues. Moreover, immunohistochemical experiments showed that Drp1, the protein expression form of DNM1L, was highly expressed in GC tissues. Next, the clinical data downloaded from the TCGA database were explored, and the expression of DNM1L was found to be significantly correlated with the T-stage of GC but not with the invasion and metastasis of GC. Next, we will use the cohort information from the tissue microarrays and the immunohistochemistry scores for a comprehensive analysis to investigate the relationship between the protein expression levels of DNM1L (Drp1) and the clinical pathological features. We found that high expression of Drp1 is significantly correlated with the T staging of gastric cancer, yet unrelated to invasion and metastasis. Furthermore, the high-expression level of Drp1 was also negatively correlated with survival among patients with GC. In order to further investigate the relationship between Drp1 expression and the prognosis of gastric cancer patients, we constructed Kaplan-Meier curves. The results showed that high DNM1L protein expression was negatively correlated with patients’ survival time. Then, by performing univariate COX regression and multivariate COX regression, DNM1L was determined to be negatively associated with patients’ prognosis and as an independent prognostic factor for shorter survival in patients with GC. These findings indicate that DNM1L positively regulates GC progression and is an independent predictor of survival in patients with GC.

Next, we conducted further research on proteins that may interact with DNM1L and the molecular mechanisms by which DNM1L may play a role in the development of GC. By taking the intersection of the GeneMANIA and STRING databases, the proteins Fis1, Mff, MIEF1, and MIEF2 were found to have a strong correlation with DNM1L. Moreover, all four proteins are involved in the recruitment of Drp1 in mitochondrial fission (27). In addition, the mitochondrial fission protein Fis1 recruits Drp1, but Fis1 and Drp1 alone may not be sufficient for mitochondrial fission (34). Mitochondrial fission factor (Mff) can activate Drp1 through its own oligomerization (35). On the contrary, the junction proteins MiD49 and MiD51 may recruit Drp1 to the mitochondrial surface independently of Fis1 and Mff (36). Through GO enrichment analysis, in BP we discovered a significant link between DNM1L and the retinoid metabolic process, highlighting its potential role in this metabolic pathway. Our findings align with recent studies that demonstrate all-trans retinoic acid (ATRA) can elevate Drp1 protein levels, subsequently promoting mitochondrial fission (37). In the CC, DNM1L is predominantly associated with skeletal muscle. The enrichment of terms such as contractile fibers, myofibrils, myonuclei, I-bands, and the cuticular envelope implies that DNM1L could be crucial for the development, maintenance, and functionality of skeletal muscle, especially in aspects related to muscle contraction and muscle structure organization. The latest research indicates that DRP1 has an important role in regulating fatty acid metabolism in skeletal muscle (38). Furthermore, in MF, DNM1L is linked to the inhibition and regulation of receptor ligands, hormones, cytokines, and diverse enzymatic activities. This suggests that DNM1L may have a substantial impact on cellular signal transduction and regulatory mechanisms. The results of KEGG enrichment analysis showed that DNM1L was significantly associated with oncogenic processes such as chemoattractant–receptor activation and chemoattractant–DNA adducts. DNM1L is also closely related to the metabolism of exogenous substances and drug metabolism of cytochrome P450 (CYP450), and mitochondria-targeted cytochrome P450 2D6 (CYP2D6) can efficiently catalyze the MPTP compounds that may induce Parkinson’s disease in humans (39). In addition, various studies have shown that CYP450 promotes angiogenesis as well as tumor growth and development by mediating the epoxidation of polyunsaturated fatty acids such as arachidonic acid (40, 41). This result suggests that DNM1L may be associated with CYP450 regulation of tumor growth. Based on the GSEA results, in the HALLMARK collection, the genes in the DNM1L high-expression group were primarily enriched in cell mitosis. For the DNM1L low-expression group, genes were enriched in immune-related activities and metabolic pathways. For the C7 set defined by MSigDB, multiple immune function gene sets were enriched in the DNM1L high and low-expression groups.

Therefore, the relationship between DNM1L expression and the level of immune infiltration in GC was investigated using the CIBERSORT algorithm, and the expression of DNM1L in the high and low groups was found to be significantly differentiated in different immune cells (plasma cells, follicular helper T cells, regulatory T cells [Tregs], γδ T cells, resting NK cells, and activated NK cells). Furthermore, the DNM1L expression was positively correlated with activated CD4 memory T cells, follicular helper T cells, and resting NK cells, as well as negatively correlated with memory B cells, plasma cells, Tregs, and activated NK cells. By taking the intersection of differential and correlation analyses, we found that DNM1L showed differences and strong correlations in plasma cells, follicular helper T cells, Tregs, resting NK cells, and activated NK cells. Recent studies have shown that the Sphk1/S1P/S1PR1 pathway enhances mitochondrial fission and increases mitochondrial mass in allogeneic CD4^+^ T cells (but not CD8^+^ T cells) through the activation of the AMPK/AKT/mTOR/Drp1 pathway (42). In addition, the downregulation of PD-1 signaling-mediated Drp1 can effectively suppress T-cell responses, and Drp1 has been proposed as a therapeutic target to improve T-cell function depleted in the fight against cancer (43). Natural killer (NK) cells play a crucial role in tumor surveillance, and the hypoxic tumor microenvironment contributes to the sustained activation of the mechanistic target of mTOR-Drp1 in NK cells, leading to the excessive fragmentation of mitochondria, and mitochondrial fragmentation prevents anti-tumor functions from occurring in NK cells (44). The abovementioned findings indicate that Drp1 plays an important role in immune infiltration and in cancer treatment.

This study has provided comprehensive understanding of the association between Drp1 and GC; however, some limitations remain. First, although the mRNA expression of Drp1 was verified on the basis of the data from the TCGA and GEO public databases and the protein expression of Drp1 by immunohistochemistry experiments, further experiments must be conducted to explore the molecular mechanisms associated with Drp1 and the mechanism by which Drp1 regulates the tumor-infiltrating cells to affect the prognosis of patients with GC. Second, the number of patients with GC, who were included in the survival analysis in the cohort of Drp1 expression, and the prognosis were small. In addition, the sample size should be increased in further studies to investigate the relationship between the prognostic value and clinicopathological features of Drp1. Third, this study only bioinformatically analyzed Drp1 for its function and value in immune infiltration, and the related metabolisms, immune pathways, and relationship with immune cells regulated by Drp1 as proposed in the article were not experimentally verified.

## 5 Conclusion

Our study demonstrated that DNM1L expression is upregulated in GC and significantly correlated with the T-stage and prognosis of patients with GC. In addition, the DNM1L differential gene set is involved in multiple metabolic and immune pathways. The expression of DNM1L was significantly correlated with the level of infiltration of multiple immune cells. Thus, DNM1L may serve as a prognostic marker for GC and may be important for immunotherapy.

## Data Availability

The original contributions presented in the study are included in the article/supplementary material. Further inquiries can be directed to the corresponding authors.
